# Phylogenetic Relationships of American Willows (*Salix* L., Salicaceae)

**DOI:** 10.1371/journal.pone.0121965

**Published:** 2015-04-16

**Authors:** Aurélien Lauron-Moreau, Frédéric E. Pitre, George W. Argus, Michel Labrecque, Luc Brouillet

**Affiliations:** 1 Institut de recherche en biologie végétale, Université de Montréal, Montréal, QC, Canada; 2 Canadian Museum of Nature, Ottawa, Canada; Field Museum of Natural History, UNITED STATES

## Abstract

*Salix* L. is the largest genus in the family Salicaceae (450 species). Several classifications have been published, but taxonomic subdivision has been under continuous revision. Our goal is to establish the phylogenetic structure of the genus using molecular data on all American willows, using three DNA markers. This complete phylogeny of American willows allows us to propose a biogeographic framework for the evolution of the genus. Material was obtained for the 122 native and introduced willow species of America. Sequences were obtained from the ITS (ribosomal nuclear DNA) and two plastid regions, *matK* and *rbcL*. Phylogenetic analyses (parsimony, maximum likelihood, Bayesian inference) were performed on the data. Geographic distribution was mapped onto the tree. The species tree provides strong support for a division of the genus into two subgenera, *Salix* and *Vetrix*. Subgenus *Salix* comprises temperate species from the Americas and Asia, and their disjunction may result from Tertiary events. Subgenus *Vetrix* is composed of boreo-arctic species of the Northern Hemisphere and their radiation may coincide with the Quaternary glaciations. Sixteen species have ambiguous positions; genetic diversity is lower in subg. *Vetrix*. A molecular phylogeny of all species of American willows has been inferred. It needs to be tested and further resolved using other molecular data. Nonetheless, the genus clearly has two clades that have distinct biogeographic patterns.

## Introduction

*Salix* L. is the largest genus of family Salicaceae with about 450 species [1–4]. The genus is distributed across the temperate to arctic regions of the Northern Hemisphere, entering tropical regions along montane ranges; willows also have been introduced worldwide. Over half the willow species, 275 are found in China [2], 107 in the former Soviet Union [5], 65 in Europe [3], and 103 in North America north of Mexico [4]. In Canada, 30% of the woody species are willows. Willows are mostly shrubs that play an important role in riparian habitats, wetlands and in shrub tundra. Willows contribute socially and economically to human societies [6–8]. During the last century, interest in environmental applications of *Salix* has grown, notably for biomass production and bioremediation [8–12].

Chase et al. [13] characterized the relationships among the genera of an expanded family Salicaceae, and Alford et al. [14] studied more closely the relationships of *Salix* and *Populus Populus* to their closest tropical and subtropical relatives. *Salix* and *Populus* are sister to each other and form a monophyletic group. In Chase et al. [13], *Itoa* and *Poliothyrsis* are successive sister to *Salix-Populus*. In Alford et al. [14], the genera *Idesia*, *Bennettiodendron* and *Olmediella* are sister to *Populus-Salix*, with *Itoa*, *Poliothyrsis*, *Carrierea* and *Macrohasseltia* sister to this clade.

**Fig 1:**
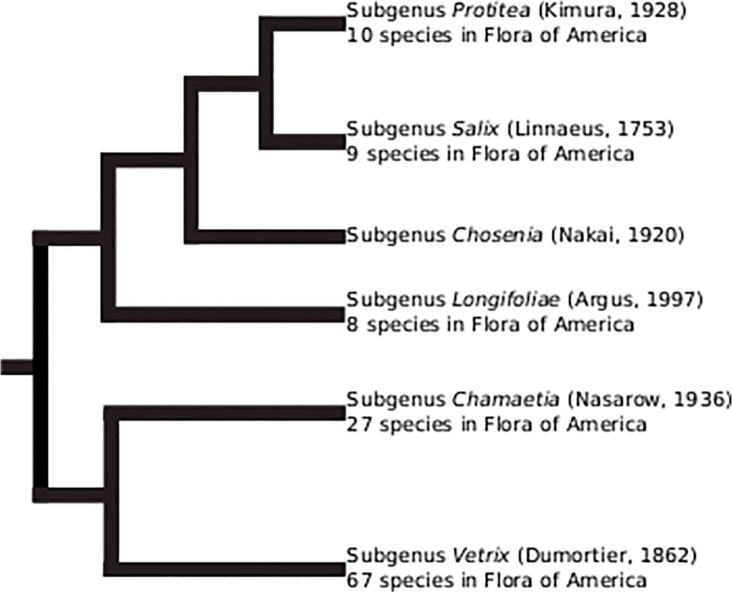
Relationships between the five subgenera of *Salix* in North America based on morphological characters (Argus, 1997). The number of species per subgenus is indicated.

Several classifications of *Salix* have been published and the subdivision of the genus has been under continuous revision. Argus [3] reviewed the history of *Salix* classifications and showed that the genus has been divided into 35 genera since its description by Linnaeus, each author using different morphologic characters to justify these divisions. For instance, some Asian treatments recognized the genera *Chosenia* Nakai [15] (*Salix arbutifolia*) and *Toisusu* Kimura [16] (*Salix cardiophylla*). Argus [3] showed that subgenus *Chosenia* (including both species above) is sister to subgenus *Salix* (Fig 1). The Angiosperm Phylogeny Group [17] included *Chosenia* and *Toisusu* within *Salix*. Several subgeneric classifications of *Salix* have been proposed. Most recently, Skvortsov [18] divided the species of the former Soviet Union and Asia into three subgenera, *Salix*, *Chamaetia* and *Vetrix*. Dorn [19] divided the American species into two subgenera, *Salix* and *Vetrix*. Based on morphology, Argus [3–4] suggested five subgenera for American willows (*Longifoliae*, *Protitea*, *Salix*, *Chamaetia* and *Vetrix*), Fig 1 illustrating the relationships between these subgenera.

Five molecular phylogenies have addressed the relationships between willow species [20–24]. Table 1 summarizes the number of species and the molecular markers used in these studies. They affirm the monophyly of *Salix*, membership of *Chosenia* (*S*. *arbutifolia*) and *Toisusu* (*S*. *cardiophylla*) within *Salix* and the presence of two major clades within the genus. These studies all included a small number of willow species relative to the total number of species in the genus.

**Table 1:** Summary of molecular phylogenies of the genus *Salix*.

Reference	Molecular region	Number of species		Main native area
		Used in study	Sequenced for study	
Leskinen and Alström-Rapaport [20]	*ITS* region	13	13	Europe
Azuma et al. [21]	*rbcL* gene	19	19	Asia
Chen et al. [22]	*rbcL* gene; *atpB-rbcL* spacer; *trnD-T* spacer	46^a^	32^b^	Asia
Hardig et al. [23]	*ITS* region; *matK* gene	25	25^c^	North America
Abdollahzadeh et al. [24]	*ITS* region; *trnL-F* region	57^d^	26^e^	Iran
This study (2014)	*ITS* region; *matK* gene; *rbcL* gene	123	122	North America

The molecular regions investigated are indicated. The number of species has been evaluated for each study (total number used and number specifically sequenced). The main native area of the species involved is indicated.

a: 45 species used with *rbcL* analysis. 31 species used in strict consensus of combined *rbcL*, *atpB-rbcL* and *trnD-T* analysis. *Salix babylonica* f. *rokkaku* was excluded from the count.

b: For 4 species, the sequences of rbcL were from Azuma et al. [21] and the spacer region was sequenced from the same specimens.

c: The sequences of *matK* for *S*. *exigua* and *S*. *interior* were from Brunsfeld et al. [76]. The *ITS* sequences of *S*. *arctica* and *S*. *discolor* are not available in GenBank.

d: *trnL-F* only for 14 species. *Salix alba* f. *alba* and *Salix* sp. (unidentified) were excluded from the count.

e: For *ITS*; only 6 species were sequenced for *trnL-F* (no analysis published). *Salix* sp. (unidentified) was excluded from the count.

The plastid genes *matK* and *rbcL*, and the nuclear ribosomal *ITS* region have been used extensively in molecular phylogenetic studies (e.g., [25–27]). Their widespread use and ease of amplification has led to their selection as the main DNA regions in the barcoding program [28–30] to be used for the identification of plants (e.g., [31]).

Our objectives are to determine the phylogenetic relationship among all American *Salix* species (107 species), using *ITS*, *matK* and *rbcL*, in order to evaluate current willow classifications and distribution patterns. We show that *Salix* is subdivided into two major clades, the first composed of temperate and the second of boreo-arctic species.

## Materials and Methods

### Plant material

This study includes all *Salix* species from America (107 species) plus the species introduced in North America (14 species: 7 from Europe, 5 from Eurasia, and 3 from Asia) [4]. The specimens were obtained from G. Argus' personal collection, the Marie Victorin Herbarium (MT), live collections of the Montreal Botanical Garden, the Canadian Museum of Nature (CAN), the Herbarium of the University of Texas (TEX), the University of Arizona Herbarium (ARIZ), and the Missouri Botanical Garden Herbarium (MO). *Chosenia arbutifolia* (= *Salix arbutifolia*) was sampled from the live collection of the Montreal Botanical Garden. A total of 213 specimens (122 species) of *Salix* were used in this study, with 1 to 3 specimens per species (S1 Table). The identity of a majority of specimens has been confirmed by G. Argus. We verified other specimens using Argus (2014). We downloaded sequences from GenBank for *Toisusu cardiophylla* (= *Salix cardiophylla*) and two outgroup genera, *Idesia* and *Populus* [13–14, 17].

### DNA extraction, amplification and sequencing

Genomic DNA was extracted from herbarium specimens or fresh leaves dried in silica gel. The CTAB method [32] was used, as modified in Lauron-Moreau et al. [33]. Three molecular regions were used in this study: *ITS*, *matK* (partial) and *rbcL* (partial). They were amplified using the specific primers detailed in Table 2. PCRs were carried out in a 20 μL solution containing 1 μL of genomic DNA (approximately 50–70 ng), 0.75X of PCR buffer (BIO BASIC, Markham, ON, Canada), 0.25 μM of each primer, 0.25 mM of dNTPs, 2.25 mM of MgCl_2_, and 1 U Taq DNA polymerase (BIO BASIC). PCRs were performed using an Eppendorf Mastercycler pro Thermal Cyclers (Eppendorf Canada, Mississauga, ON, Canada) under the following cycling parameters: initial denaturation at 94°C for 3 min followed by 33–35 cycles (C_a_) of 30 s at 94°C, 30 s at 52°C, 45–70 s at 72°C; and followed by a final extension at 72°C for 5 min. PCR products were sequenced by the group McGill University and Génome Québec Innovation Centre. Over half the sequences of *matK* and *rbcL* were obtained with the help of the Barcode of Life Data Systems (BOLD) following standard protocols at the Canadian Centre for DNA barcoding (CCDB) for plants, as described in Kuzmina et al. [31].

**Table 2:** Primers and PCR cycle characteristics for the three genes used in the study, indicating: source of primers; number of sequences obtained and their length (bp); percentage of polymorphic sites (including and excluding outgroup).

ene	Source	Primer name	Sequence 5'-3'	Number of cycles (C_a_)	Elongation time (s)	Number of sequences	Alignment length (bp)	Polymorphic sites	
								With outgtoup	Without outgroup
*ITS*	this paper	ALM-P001	F: CGTAACAAGGTTTCCGTAGG	35	60	210	608	125 (21%)	95 (16%)
		ALM-P002	R: TGCTTAAACTCAGCGGGTAG						
*matK*	Ford et al. [77]	matK X	F: TAATTTACGATCAATTCATTC	33	70	212	874	106 (12%)	50 (6%)
	Kew Barcoding	matK_Equisetum	R: GTACTTTTATGTTTACGAGC						
*rbcL*	Levin et al. [78]	P1630	F: ATGTCACCACAAACAGAGACTAAAGC	33	45	212	553	44 (8%)	20 (4%)
	Kress et al. [79]	rbcLa-R	R: GTAAAATCAAGTCCACCRCG						

### Sequence alignment and phylogenetic analyses

Sequences were assembled using Geneious Pro version 4.8.5 created by Biomatters (http://www.geneious.com). Alignments were done in SeaView version 4.2.6 [34] using Muscle parameters [35], followed by manual correction. Parsimony, maximum likelihood (ML), and Bayesian (BA) analyses were performed to determine the phylogenetic relationships on four datasets: *ITS*, *matK*, *rbcL*, and the concatenated *matK*-*rbcL* sequences. The program jModelTest2 [36–37] was used to select the model of sequence evolution for ML and BA analyses. Data matrices from this study are available on TreeBase (website: http://treebase.org) by searching for study ID14313.

Parsimony analyses were performed using PAUP version 4.0b10 [38]. We selected the optimal trees using a heuristic search following these parameters: 100 random additions of sequences followed by tree bisection and reconnection (TBR) branchswapping, retaining at most 100 trees at each replicate. Branch support was estimated using 10,000 bootstrap replicates with the same heuristic settings.

Maximum likelihood analyses were performed using PhyML 3.0 [39]. ML heuristic searches and bootstrap analysis (10,000 replicates) were conducted to obtain the best trees under the parameters of the evolution model selected by jModelTest2. The adequate evolution models were GTR+G+I for *ITS*, GTR+G for *matK* and *matk*-*rbcL*, and K80+I for *rbcL*.

Bayesian analyses were performed using MrBayes version 3.1.2 [40] and BEAST v1.7.5 [41]. In MrBayes, two independent runs were performed, each consisting of four parallel Markov chain Monte Carlo (MCMC) of 100 million generations (the average standard deviation of split frequencies being lower than 0.01). Trees were sampled every 10,000 generations. The evolution models used were identical with those in the ML analyses. Tree parameters reached stationarity after a burn-in period of 250,000 generations. Optimal trees were then sampled every 1,000 generations to obtain the final consensus tree and associated posterior probabilities. For the BEAST analysis, each molecular region was analyzed separately and the species tree was developed concurrently. Two independent runs of 100 million generations were performed, each with sampling every 10,000 generations. We used the same evolution models as above, with four gamma categories, a coalescent tree prior and a strict clock model for each partition. After analysis, the software Tracer [42] was used to evaluate the convergence after the first 20% of generations had been discounted as burn-in. The software TreeAnnotator v1.7.5 (available in BEAST package) was used to estimate the maximum-clade-credibility using the Bayesian posterior probabilities.

On the BEAST species tree, we illustrated the main native area of each species following Argus ([43]; for North America), using seven zones: four in North America (western temperate, western boreo-arctic, eastern temperate, eastern boreo-arctic) and three representing Europe, Asia and Mexico (including Central and South America). Finally, we constrained a BEAST analysis to conform with the morphological classification of Argus [3].

## Results and Discussion

### Success rate of the amplifications and DNA sequences

We obtained 211 sequences for *ITS* (including 2 partial sequences) and 213 sequences for *matK* and *rbcL* (all sequences are available in GenBank) (S1 Table). For the *ITS* region, amplification of two specimens of *S*. *atrocinerea* was not a successful, and amplification was partial only for *S*. *jaliscana* and *S*. *prolixa*. New DNA extractions and a modification to the PCR protocol did not give better results. Fifteen sequences were downloaded from GenBank and aligned with our data. We did not find intra-species variation within our data. The alignment of *ITS*, *matK* and *rbcL* resulted in 608, 874 and 553 aligned nucleotides, respectively (Table 2). Including the GenBank data, we had 215 sequences for *ITS*, and 217 sequences for *matK* and *rbcL*. The *ITS* region had a higher proportion of polymorphic sites (21%) when compared with *matK* (12%) and *rbcL* (8%) (Table 2).

### Polymorphisms

We observed many polymorphic sites in the *ITS* region. Sixteen species (*S*. *arbusculoides*, *S*. *arctica*, *S*. *arctophila*, *S*. *barclayi*, *S*. *cana*, *S*. *columbiana*, *S*. *discolor*, *S*. *exigua*, *S*. *famelica*, *S*. *floridana*, *S*. *humboldtiana*, *S*. *jejuna*, *S*. *monticola*, *S*. *raupii*, *S*. *richardsonii*, *S*. *rotundifolia*) had polymorphisms at 17 nucleotide sites (1–5 polymorphic sites per species). We also found polymorphisms in the plastid genes. *Salix aeruginosa* and *S*. *jaliscana* are polymorphic at four (34, 367–368, 398) and two sites (80, 514), respectively, in *matK*. Six species had polymorphic sites on *rbcL*: *S*. *jaliscana* (396–397); *S*. *pedicellaris*, *S*. *pseudomyrsinites* (285); and *S*. *argyrocarpa*, *S*. *cascadensis*, *S*. *orestera* (286).

### Phylogenetic analyses

**Fig 2:**
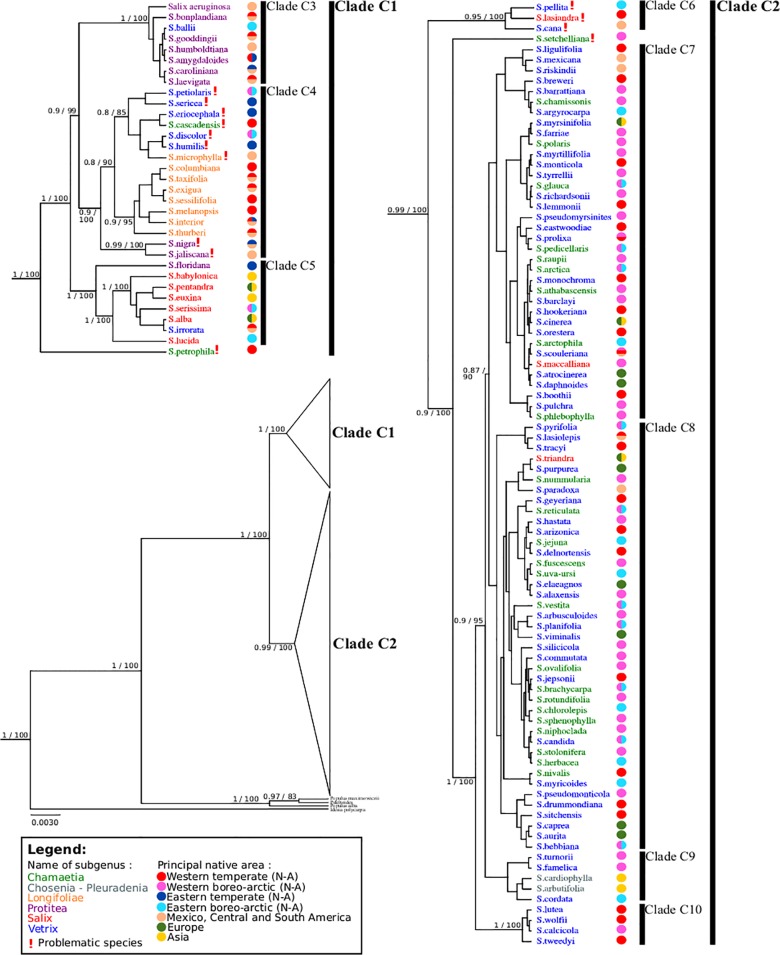
BEAST gene tree of *matK* and *rbcL*. Branch support is Bayesian posterior probabilities and ML bootstrap values; subgenera are identified using colors; *Idesia* and *Populus* are outgroups.

**Fig 3:**
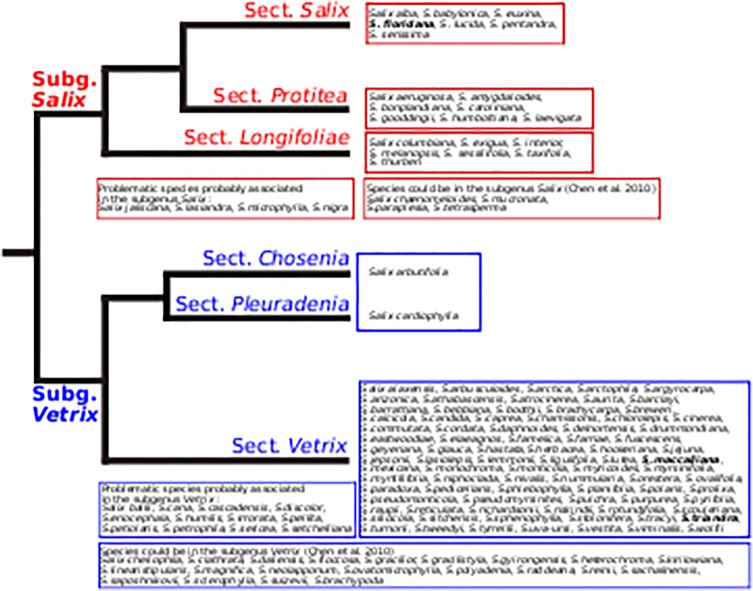
BEAST gene tree of *ITS*. Branch support is Bayesian posterior probabilities and ML bootstrap values; subgenera are identified using colors; *Idesia* and *Populus* are outgroups.

We compared the resolution and branch support of four analytical approaches (PhylML, MrBayes, BEAST and PAUP) on all datasets. The topologies were similar and we are presenting the results from BEAST because its support values were higher (Figs 2 and 3). Bayesian posterior probabilities and ML bootstrap values are provided on the trees shown.

The phylogenetic trees obtained for *matK* and *rbcL* were identical except for the position of *Salix petrophila*, and we are presenting the consensus tree of these two plastid genes (Fig 2). Two major clades are apparent on the cp DNA tree. Clade A1 includes 32 *Salix* species and clade A2, 88. The two clades are well supported. The relationships within each clade are not well resolved, however, and branches with a posterior probability lower than 0.7 were collapsed. Clade A1 comprises the majority of species from subgenera *Longifoliae*, *Protitea* and *Salix*. Clade A2 includes most species of the subgenera *Chamaetia* and *Vetrix*. *Salix arbutifolia* and *S*. *cardiophylla* belong to clade A2.

In the *ITS* tree, four different crown are well supported (Fig 3). Clade B1 comprises most species of subgenus *Protitea*, clade B2 most species of subgenus *Salix*, clade B3 most species of subgenus *Longifoliae*, and clade B4 most species of subgenera *Chamaetia* and *Vetrix*. Overall, the species of clades B1, B2 and B3 (Fig 3) are present in clade A1 of the cp trees (Fig 2), while B4, *S*. *arbutifolia* and *S*. *cardiophylla* are in clade A2. Fourteen taxa have incongruent positions on the two trees, however.

**Fig 4:**
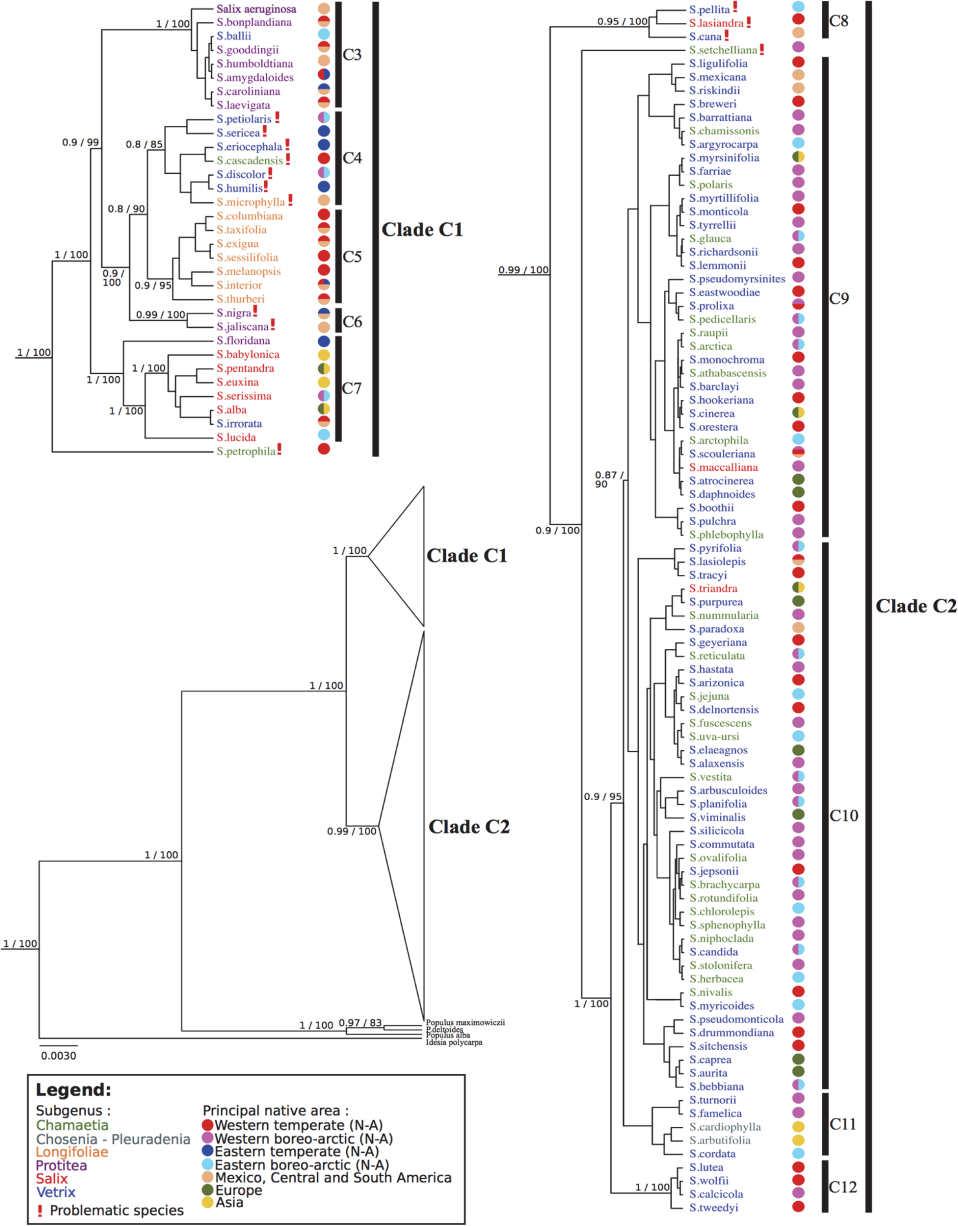
BEAST species tree generated with *ITS*, *matK* and *rbcL*. Branch support is Bayesian posterior probabilities and ML bootstrap values; subgenera are identified using colors; native areas (Argus 2007) are indicated by colored circles; *Idesia* and *Populus* are outgroups.

Fig 4 presents the species tree of the simultaneous BEAST analysis of the three markers. The tree exhibits two major clades, C1 and C2. Many subclades are shown in clade C1 and in clade C2. Subclades C9 or C10 have low support. The distribution of species is presented on the tree.

S1 Fig shows a BEAST analysis where the subgenera were constrained according to Argus [4]. Support is slightly lower than Fig 2. In a manner similar to the unconstrained BEAST tree (Fig 4), one clade includes the species of the subgenera *Longifoliae*, *Protitea* and *Salix*, and a second one, the species of subgenera *Chamaetia* and *Vetrix* plus *S*. *arbutifolia* and *S*. *cardiophylla*. Species that in Fig 4 did not group with the subgenus in which Argus classified it all diverge early in the clades of the constrained tree.

### Sequence polymorphism

As in Leskinen and Alström-Rapaport [20], we observed intra-individual polymorphic nucleotide sites in the *ITS* region. Ribosomal sequences are present in thousands of copies in the nuclear genome [44]. Usually, sequences within individuals are uniformized due to concerted evolution [45–46]. However, in cases of recent hybridization or homoploid speciation, sequence homogenization is often not achieved in the short period of time involved [45–46]. For instance, Leskinen and Alström-Rapaport [20] hypothesized that *S*. *schwerinii* could result from homoploid speciation after hybridization between *S*. *viminalis* and a second, unidentified species. In our study, *S*. *exigua*, a diploid species, shows polymorphic sites. It could be due either to introgression from recent hybridization or the species could be the result of homoploid speciation. Other species, however, are polyploid and polymorphism may merely result from a lack of homogenization, particularly in allopolyploid taxa. Conversely, *Salix alba*, a tetraploid, is without polymorphic sites in the *ITS* region. Data are currently insufficient to explain the presence of polymorphic sites in the *ITS* of American *Salix*.

Polymorphic nucleotide sites in plastid sequences may seem surprising but are not new. Few studies have reported this [47–48]. One hypothesis to explain such polymorphism would be the inclusion of cp DNA fragments in nuclear DNA [48–49]. A second hypothesis would be an error occurring during plastid division [50]. Our data are insufficient to determine what mechanism is acting. It would require, among others, detailed population and genomic studies of the species concerned.

### Phylogenetic relationships between American species of willows

#### Plastid trees

The plastid tree (Fig 2), using *Populus* and *Idesia* as outgroups, affirms the monophyly of genus *Salix* and the inclusion of *S*. *arbutifolia* (*Chosenia*) and *S*. *cardiophylla* (*Toisusu*) within the genus, as was shown by Chen et al. [22]. This tree also shows the separation of American *Salix* species into two major clades, as was also found by Azuma et al. [21] and Chen et al. [22] on Asian species (see Table 1). As in our study, one clade included species of subgenera *Chamaetia* and *Vetrix* (our A2 clade), and the other (our clade A1) subgenus *Salix* (no representative of subg. *Longifoliae* and *Protitea* were included). Twenty species were shared between our study and that of Chen et al. [22], 18 of which are found in the same clade in both analyses (shown by black stars in Fig 2). Two taxa, *S*. *discolor* and *S*. *maccaliana*, were found in different clades, however. This could be explained by the fact that the two species are polyploid, 4x and 10x, respectively [4]; it could also be the result of intra-specific variability or chloroplast capture following hybridization, or of an error of identification or manipulation. Despite differences possibly caused by the taxonomy used (see below), Hardig et al. [23], working on American species (Table 1), also retrieved two similar clades. Resolution within clades is low and poorly supported, resulting in polytomies. Low rates of evolution of the plastid genome or recency of speciation in willow species could explain this. The positions of *S*. *petrophila* (clade A1) and *S*. *lasiandra* (clade A2) are surprising, both being early divergent in each clade; this cannot be readily explained with current data.

#### ITS tree

The *ITS* region shows more variation than the cp DNA markers, but resolution of the tree was not greatly improved. *ITS* trees in Leskinen and Alström-Rapaport [20], Hardig et al. [23] and Abdollahzedeh et al. [24], built respectively using parsimony, maximum likelihood and MrBayes, were similar to our analyses (not shown) carried out with the same approaches: a large polytomy is retrieved, with a single small clade comprised of species belonging to subg. *Salix*, *Longifolia* and *Protitea*. Our BEAST tree (Fig 3), however, provided greater resolution, identifying four clades: species of subgenus *Protitea* in clade B1, subgenus *Salix* in B2, and subgenus *Longifoliae* in B3, with subgenera *Chamaetia* and *Vetrix* species intermixed in the large clade B4. All North American species used by Hardig et al. [23] were also included in our study (shown by black stars in Fig 3). Differences were observed in the placement of a few species. For instance, Hardig et al. included specimens of *S*. *eriocephala* and *S*. *lucida* from Idaho, species that Argus [4] do not report for this area; this may result from the taxonomy used, since varieties sometimes attributed to *S*. *eriocephala* in western North America, for instance, are considered distinct species by Argus.

#### Incongruence between the chloroplastic and ITS trees

Overall, clades A2 (cp) and B4 (*ITS*) include species of subg. *Chamaetia* and *Vetrix*, while the species of clade A1 (cp) coincide with those in clades B1, B2 and B3 (*ITS*) (Figs 2 and 3). The global structure of the trees is similar. There is significant incongruence however, for 14 taxa. Ten species of clade A1 in the cp tree (Fig 2) were found in clade B4 on the *ITS* tree (Fig 3). Conversely, four species of clade A2 (cp) were retrieved in clade B4. These taxa are highlighted in our trees. Five hypotheses could explain these incongruences. Firstly, plastid or rDNA capture following hybridization could have occurred. For instance, *S*. *pellita* (clade B4) can form natural hybrids in nature with *S*. *alaxensis* (clade B1) (Argus, 2014). Secondly, part of the chloroplast genome of one parent could have migrated to the nucleus in allopolyploid taxa, a rare but not impossible phenomenon [51]. Thirdly, horizontal gene transfer from another species is possible [52]. Fourth, plastid fusion may occur, though it is rarely documented [53]. And finally, field or laboratory errors could have happened, which seems improbable given the number of taxa involved.

#### Species tree

The species tree (Fig 4) results from the simultaneous BEAST analysis of the cpDNA and *ITS* datasets. More resolution is apparent on this tree. The topology affirms the presence of two major clades (C1 and C2), such as described above, which mirrors the tree inferred from plastid data but is incongruent (in part) with the tree inferred from nuclear data. Within clade C1, species are mostly grouped according to the subgenera where they are assigned by Argus [4], i.e., subg. *Longifoliae* (subclade C5), *Protitea* (subclade C3), and *Salix* (subclade C7, also retrieved in Chen et al. [22]), which are well supported. A few species (discussed below) appear in novel positions with respect to Argus [4]. Subgenera *Chamaetia* and *Vetrix* (clade C2) form one group, which corresponds to the observations of Chen et al. [22]. There is no clear pattern of subgeneric segregation in clade C2. All subclades within C2 have low support. Skvortsov [18], discussing Russian material, also indicated that the distinction between these subgenera was difficult, while Dorn [19] only recognized subg. *Vetrix*. The branching order and groupings observed in this analysis are similar to those obtained in a morphology-based, numerical analysis of *Salix* by Argus [3], if one excepts the position of subg. *Chosenia*, which groups in C2 in our analysis and the equivalent of C1 in that of Argus.

Fourteen species had different positions in the *ITS* and cp DNA analyses. They are indicated by a! in Figs 2–4. Seven species, *Salix cascadensis*, *S*. *discolor*, *S*. *eriocephala*, *S*. *humilis*, *S*. *microphylla*, *S*. *petiolaris* and *S*. *sericea*, form a subclade (C4) within clade C1 in the analysis. Yet, the morphology of these species is heterogeneous [4] and no morphologic character appears to support such a group. Also in clade C1, *S*. *jaliscana* and *S*. *nigra* are grouped, both of which belong to subg. *Protitea* (Argus [4]), which would make subg. *Protitea* paraphyletic to subg. *Longifoliae* and the artificial subclade C4. All these species were in clade A1 in the cp tree and in clade B2 in the *ITS* tree. The grouping of species in subclade C4 suggests a random grouping of species with similar behaviors. Subclade C4 appears artificial. *Salix petrophila* (subg. *Chamaetia*) appears to be an early diverging branch of clade C1. This species occupied different positions in the *matK* and *rbcL* trees (not shown). Similarly, clade C6, comprised of *S*. *cana*, *S*. *lasiandra*, and *S*. *pellita*, form an early diverging group sister to clade C2. *Salix setcheliana* is an early diverging branch within clade C2. All these species were in clade A2 in the cp tree and in clade B1 in the *ITS* tree. In all instances, it appears as if the position in the species tree is determined primarily by the cp DNA.

#### Tree constrained by subgenera

A BEAST analysis of all datasets was done while constraining species to their subgeneric affiliation (fide Argus, [4]) (S1 Fig). Support for the subgenera in this tree is lower than for clades and subclades in the unconstrained species tree, where some subclades roughly correspond to subgenera. The constrained tree is divided into two clades, as in the unconstrained species tree. Subgenera *Chamaetia* and *Vetrix* are sisters within one clade, and subg. *Longifoliae*, *Protitea* and *Salix* are grouped in the second. Five species, however, that belong to a particular subgenus in the constrained tree, occupy a different position in the species tree. This cannot be explained readily by chloroplast capture, introgression or hybridization between *Salix* species. The 14 problematic taxa considered above, when constrained to group with their subgenus, acquire a basal position in their subgenus, which casts doubt as to their membership. Constraining the analysis to respect the subgenera defined by Argus [3–4] assumes that the morphological characters used are proper to classify willow species. The species tree (Fig 4), however, indicates that this is not valid for all characters and species, particularly when one considers the distinction between subg. *Chamaetia* and *Vetrix*.

#### Subgenus attribution of three species

The species tree (Fig 4) shows that three species, *Salix floridana* (subg. *Protitea*), *S*. *maccalliana* and *S*. *triandra* (both subg. *Salix*), probably are assigned to the wrong subgenus. Our data suggest that *S*. *floridana* belongs to subgenus *Salix*, where it would be sister to the other species. Chen et al. [22] also found a similar position for *S*. *floridana*. The composition of subg. *Protitea* (*S*. *amygdaloides*, *S*. *bonplandiana*, *S*. *caroliniana*, *S*. *gooddingii*, *S*. *humboltiana*) has been discussed repeatedly [54–56], without consensus. Dorn [19] proposed the exclusion of *S*. *floridana* from this subgenus, placing it instead in either subg. *Salix* or *Vetrix*. He hypothesized that the morphological similarities (bud scales distinct, flowers with 3 to 7 stamens) of this species to subgenus *Protitea* was the result of hybridization [19]. Argus [4, 57] classified species of subgenus *Protitea* together because they share many morphological traits. The branching of the species tree (Fig 4) suggests that the morphological similarities highlighted by Dorn could be symplesiomorphic and not the result of hybridization. Chmelar [58] proposed that ovule number could be taxonomically significant. *Salix floridana* and *S*. *babylonica* have 2 ovules per carpel, *S*. *alba* 3 to 6, and *S*. *amygdaloides*, *S*. *caroliniana* and *S*. *nigra* 6 to 9 [57]. Low ovule number could be a feature of sect. *Salix*.

Our data suggest that *S*. *maccalliana* and *S*. *triandra* belong in a large subgenus *Vetrix* (see below). *Salix maccalliana* is decaploid or dodecaploid [4], which indicates a complex origin. Its morphology is similar to that of *S*. *lucida* (subg. *Salix*) [4]. The staminate flowers with abaxial nectaries and tawny, persistent bracts, and the villous ovaries, however, suggest relationship with *S*. *glauca* (subg. *Chamaetia*). Dorn [19] placed this species in subgenus *Vetrix*. Chen et al. [22] included *S*. *maccalliana* in subgenus *Salix*. The provenance of their sample appears geographically suspect, however. In the case of *S*. *triandra*, both our study and those of Leskinen and Alström-Rapaport [20] and Chen et al. [22] that it belongs with subg. *Vetrix*. The latter fully discussed this issue and indicated that *S*. *triandra* could be considered to belong to a distinct subgenus. In our tree (Fig 4), however, *S*. *triandra* falls fully within subg. *Vetrix*.

#### Placement of *Salix ballii* and *Salix irrorata*

*Salix ballii* and *S*. *irrorata* are assigned to subgenus *Vetrix* by Argus [4]. Our data, however, show that *S*. *ballii* is related to subg. *Protitea*, and *S*. *irrorata* to subg. *Salix*. Two distinct specimens were sequenced for each species with the same result. One hypothesis would be the capture of plastid or ribosomal DNA after hybridization. A laboratory error cannot be excluded. At this time, data are insufficient to explain these placements.

### Biogeography of *Salix*

Formal biogeographic analyses (DEC [59], or DIVA [60]) could not be carried out with our tree due notably to the lack of resolution in clade C2 (Fig 4). Doing a calibrated datation was not feasible at this time due to a lack of verified *Salix* fossil material that could be accurately placed on the tree topology. Nonetheless, the patterns observed on Fig 4 allow the formulation of biogeographic hypotheses.

Overall, most species of clade C1 are found in temperate regions of both North America (western and eastern) and Eurasia, while those of clade C2 include mostly species from boreo-arctic regions or montane areas southward. Globally, distribution patterns in *Salix* reflect well the biogeographic regions delimited by Takhtajan [61] within the Holarctic kingdom, Boreal subkingdom: C1 taxa are predominantly in the Eastern Asiatic, North American Atlantic and Rocky Mountain regions (= subg. *Salix*, see below), and C2 taxa in the Circumboreal region (= subg. *Vetrix*, see below).

Within C1, species of subclade C3 are western and eastern North American, with extensions to Mexico and Central to South America (*S*. *humboldtiana* only). Most species of subclade C5 are temperate western North American and Mexican (*S*. *interior* spreading to eastern North America). Within subclade C7, *Salix floridana* is in southeastern temperate America (mostly Florida) and is sister to a clade comprised of temperate Eurasian and American elements. Subclade C4, which includes problematic elements, is predominantly eastern North American with some elements more boreal and therefore more widespread. We hypothesize that inter-continental migrations between temperate regions during the Tertiary may explain the pattern observed. During the Tertiary, the North Atlantic and Bering land bridges [62–64] allowed inter-continental exchanges at high latitudes because of warmer climates [65]; such a disjunction pattern appears to fit distributions in clade C1, and has been documented and dated for other taxa in the Northern Hemisphere (e.g. [66–69]).

Clade C2 comprises more northern, boreo-arctic and montane to alpine species of both Eurasia and North America; all strictly European species appear to belong here. North American and Eurasian elements are intermixed throughout the tree, as are western and eastern North American boreal species. A few Mexican species (*S*. *cana*, *S*. *mexicana*, *S*. *riskindii*, *and S*. *paradoxa*), closely related to western North American montane species, are found here, as are species found in both Mexico and western North America (*S*. *lasiolepis*, *S*. *scouleriana*). In Asia, *S*. *cardiophylla* (*Toisusu*) is montane and cool temperate, and *S*. *arbutifolia* (*Chosenia*) is montane and boreo-temperate; biogeographically, they fit well within this clade. We hypothesize that the large radiation within clade C2 is the result of events that occurred during the Pleistocene. Several species in subg. *Vetrix* are circumarctic, widespread at boreal latitudes, amphi-Beringian or amphi-Atlantic. During this period, the Northern Hemisphere was subjected to several glaciation periods [70–71].

Notably, lowering of the sea during glacial events repeatedly opened the Bering land bridge for long periods of time, allowing migration at high latitudes in a corridor of steppe and tundra. Ice extension also forced boreo-arctic species to seek refuges south of the ice. Both phenomena impacted species distributions [72]. These migrations brought species in contact and favored hybridization and the formation of polyploid species (e.g., [73]), two factors that appear to have played an important role in the rapid diversification of subg. *Vetrix*.

### Classification of *Salix*

Skvortsov [18] divided the species of the former Soviet Union and Asia into three subgenera, Dorn [19] the American species into two subgenera, and Argus [3–4] American taxa into five subgenera (*Longifoliae*, *Protitea*, *Salix*, *Chamaetia* and *Vetrix*). Our molecular phylogenetic study and that of Chen et al. [22] show a primary subdivision of *Salix* into two clades (Fig 4), the latter pointing out that the number of subgenera proposed for *Salix* was too high. The studies by Leskinen and Alström-Rapaport [20], Azuma et al. [21], Hardig et al. [23] and Abdollahzedeh et al. [24] also suggest such a division. We are proposing to divide *Salix* into two subgenera, *Salix* and *Vetrix*. Three sections may be recognized within subgenus *Salix*: *Salix*, *Protitea*, and *Longifoliae*, the latter American only. Within subgenus *Vetrix*, lack of resolution prevents the definition of sections at this time. *Salix arbutifolia* (*Chosenia*) and *S*. *cardiophylla* (*Toisusu*) are definitely members of subg. *Vetrix*, possibly as an early branch including other willows, a group that may deserve sectional recognition. Another unresolved issue is the definitive position of the 16 problematic species discussed above, notably the clade within subg. *Salix*.

The three molecular regions used in our study are the markers selected for the barcoding of plants. The degree of variation of these molecular markers in *Salix*, however, is insufficient to provide species identification in subg. *Vetrix*, and as other studies have shown [74], other regions will need to be developed for full barcoding of willows.

### Conclusions

We present the first complete phylogeny of willows for the Americas, based on three molecular markers from plastid and nuclear ribosomal DNA. We affirm the subdivision of genus *Salix* species into two clades that correspond to two subgenera proposed earlier on the basis of morphologic and molecular studies. Nonetheless, relationships among species remain tentative due to a lack of resolution within subg. *Vetrix* and to the unusual relationships exhibited by a 16 problematic species. Further phylogenetic analyses using low-copy nuclear genes should help address this lack of resolution and membership issues, and help in obtaining a tree that could be the object of formal biogeographic analyses. The challenge presented in this genus by hybridization and polyploidy may be resolved by phylogeographic analyses of species complex, such as was done by Tsai and Carstens [75]. In a recent report (2014), Percy et al. described the difficulty of DNA fingerprinting for willow species when using only plastid regions because these markers are unable to delineate possible widespread hybridization events, thus supporting our choice to include a nuclear marker [80].

Our phylogenetic analysis provides a framework to interpret data from other fields of study, such as eco-physiology and the development of willows for economic usages, such as biomass production.

## Supporting Information

S1 Fig BEAST species tree generated with *ITS*, *matK* and *rbcL*, constrained to fit the subgenera of Argus (2010). Branch support is Bayesian posterior probabilities and ML bootstrap values.
(TIF)

S1 Table Willow species *(Salix* L.) used in this study. We indicated species name (according in Argus [4] and IPNI), their status in America (native or introduced), their principal native area, their subgenus (Argus [4]), the herbarium informations: live collections of the Montreal Botanical Garden (MBG), the Canadian Museum of Nature (CAN), the Herbarium of the University of Texas (TEX), the University of Arizona Herbarium (ARIZ), the Missouri Botanical Garden Herbarium (MO), or unmounted. Finally, we have indicated the GenBank number.
(DOCX)
