# Correction: Phylogenetic Relationships of American Willows (*Salix* L., Salicaceae)

**DOI:** 10.1371/journal.pone.0138963

**Published:** 2015-09-18

**Authors:** Aurélien Lauron-Moreau, Frédéric E. Pitre, George W. Argus, Michel Labrecque, Luc Brouillet

Previous revision versions of Fig 2, Fig 3, and S1 Fig were incorrectly published. Please view the correct Fig 2, Fig 3, and S1 Fig here.

**Fig 2:**
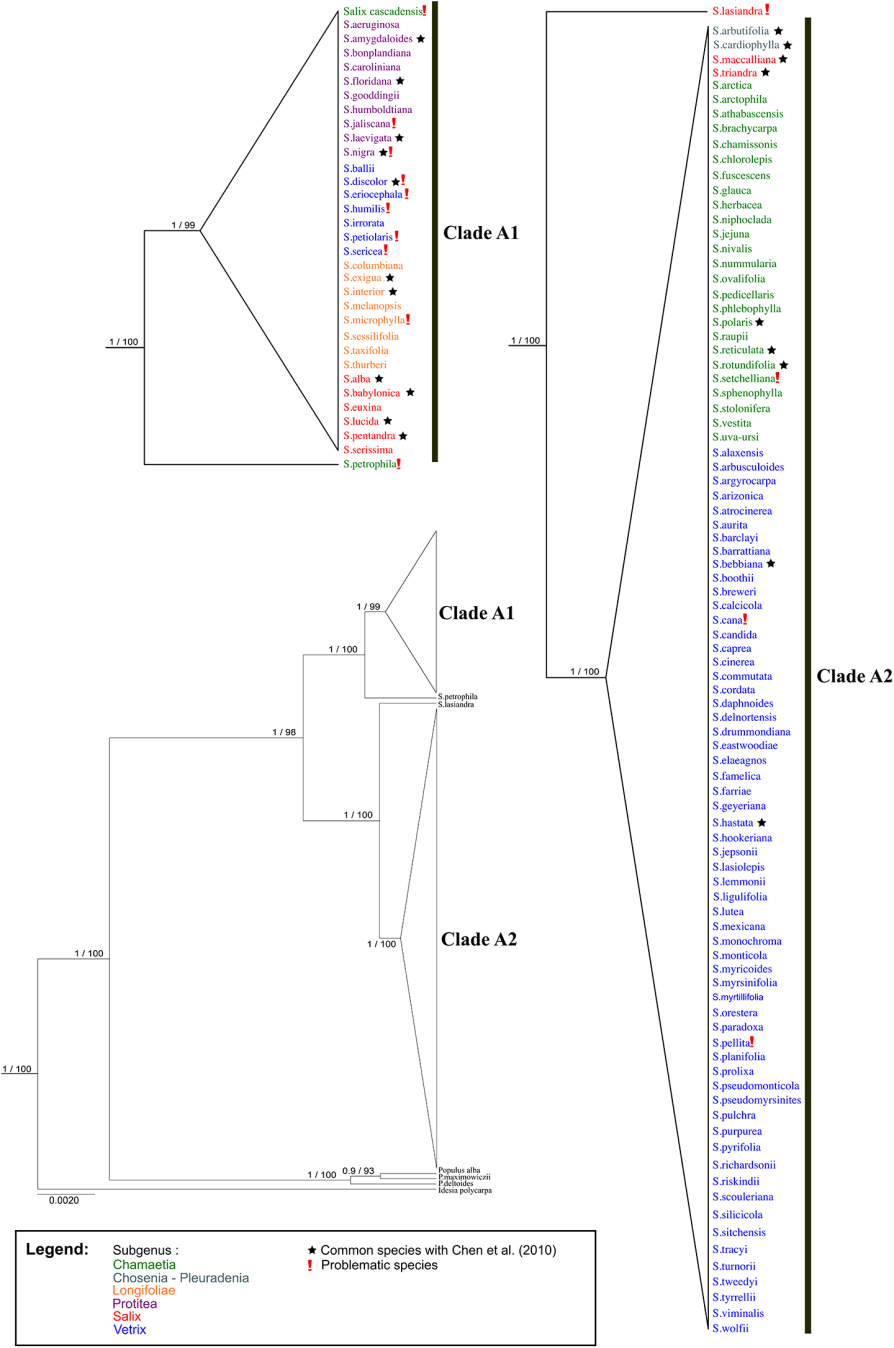
BEAST gene tree of *matK* and *rbcL*. Branch support is Bayesian posterior probabilities and ML bootstrap values; subgenera are identified using colors; *Idesia* and *Populus* are outgroups.

**Fig 3:**
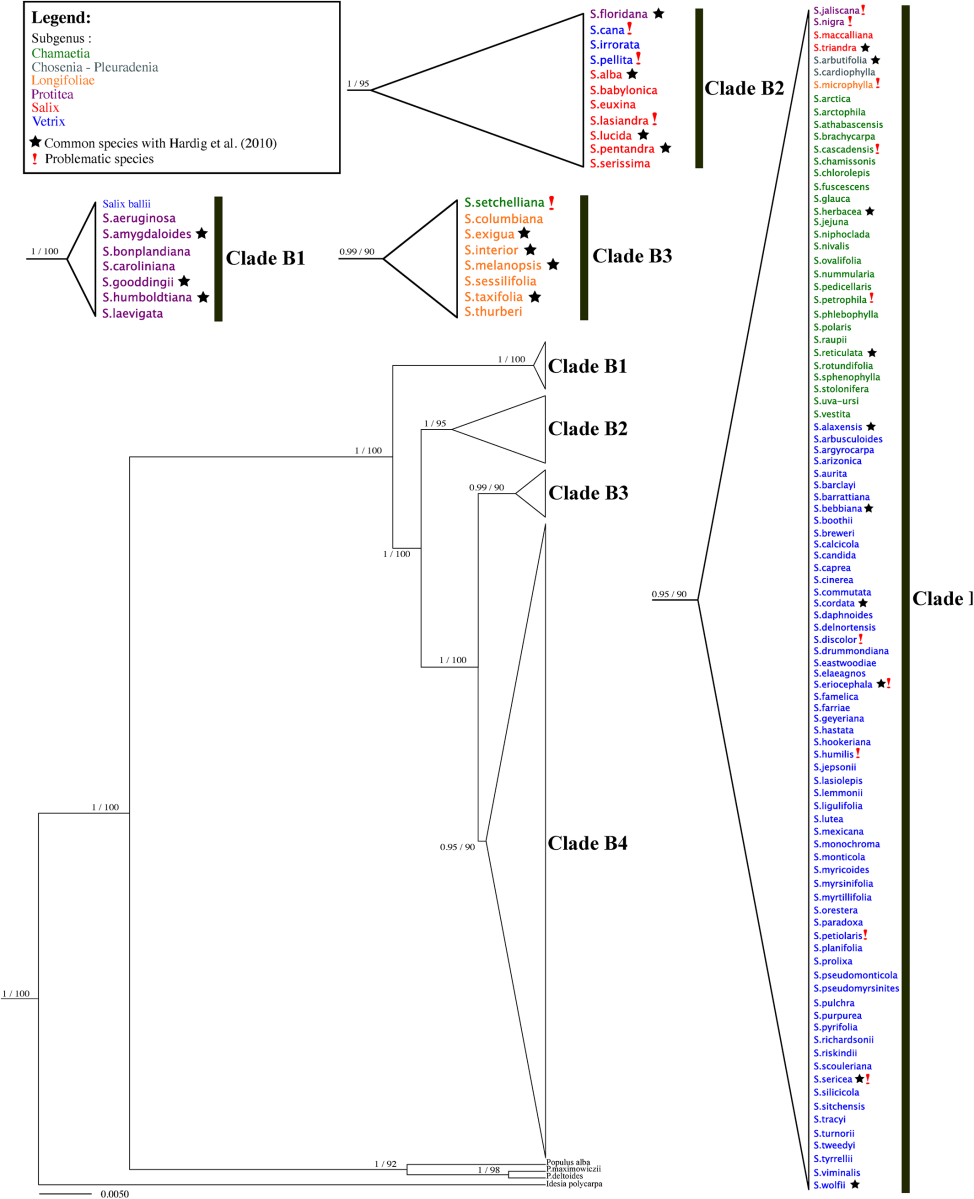
BEAST gene tree of *ITS*. Branch support is Bayesian posterior probabilities and ML bootstrap values; subgenera are identified using colors; *Idesia* and *Populus* are outgroups.

## Supporting Information

S1 Fig BEAST species tree generated with *ITS*, *matK* and *rbcL*, constrained to fit the subgenera of Argus (2010). Branch support is Bayesian posterior probabilities and ML bootstrap values.
(EPS)
