# Supplementary figures and images for: Phylogenetic Relationships of American Willows (Salix L., Salicaceae)
Source: PLoS One. 2015 Apr 16;10(4):e0121965. doi: 10.1371/journal.pone.0121965 (PMC4399884; doi:10.1371/journal.pone.0121965)

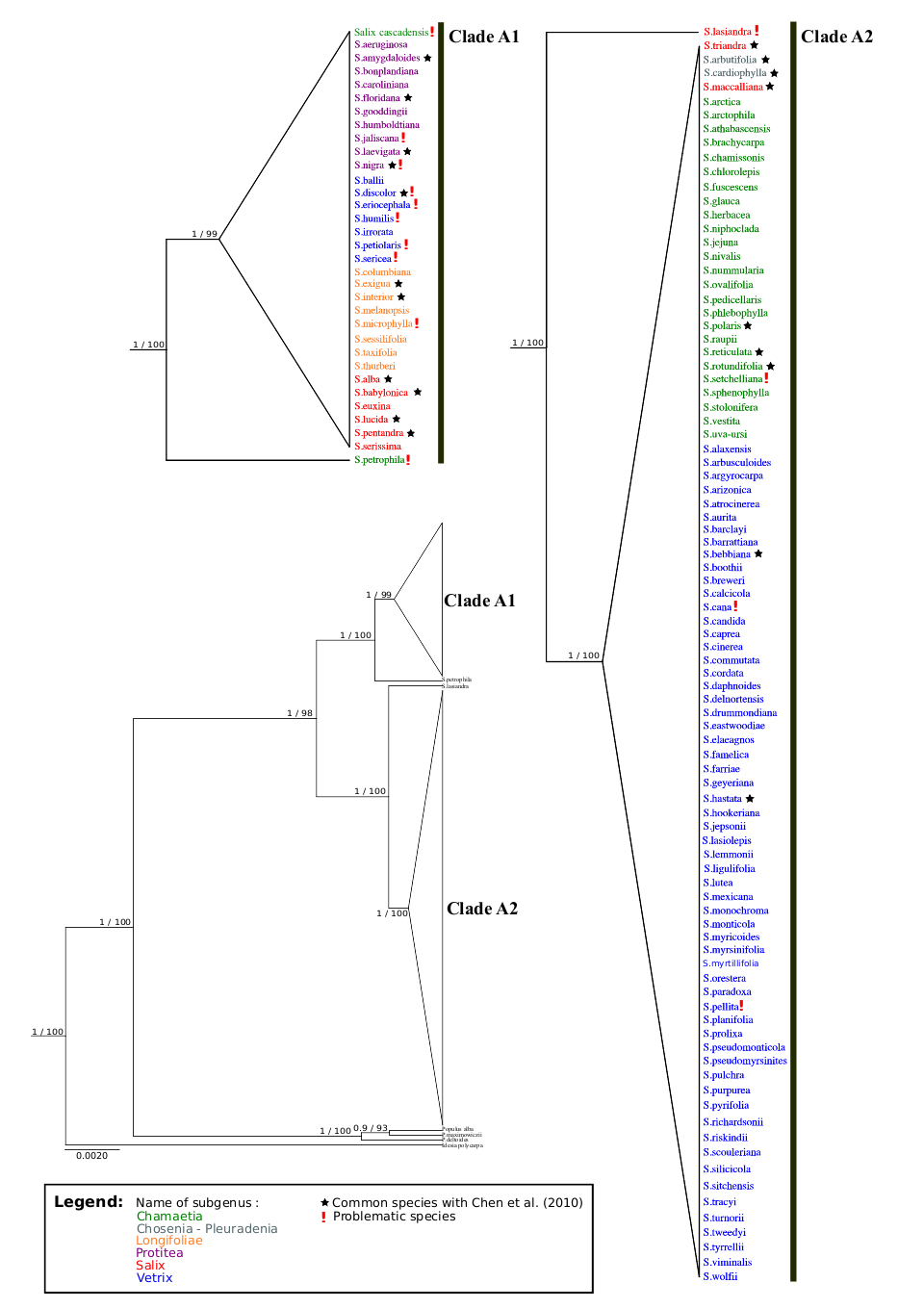

Supplement: S1 Fig — Branch support is Bayesian posterior probabilities and ML bootstrap values. (TIF) [file pone.0121965.s001.TIF]
