# Supplementary material for: Phylogenetic Relationships of American Willows (Salix L., Salicaceae)
Source: PLoS One. 2015 Apr 16;10(4):e0121965. doi: 10.1371/journal.pone.0121965 (PMC4399884; doi:10.1371/journal.pone.0121965)
Supplement: S1 Table — We indicated species name (according in Argus [4] and IPNI), their status in America (native or introduced), their principal native area, their subgenus (Argus [4]), the herbarium informations: live collections of the Montreal Botanical Garden (MBG), the Canadian Museum of Nature (CAN), the Herbarium of the University of Texas (TEX), the University of Arizona Herbarium (ARIZ), the Missouri Botanical Garden Herbarium (MO), or unmounted. Finally, we have indicated the GenBank number. (DOCX) [file pone.0121965.s002.docx]

| **Table S1**. Willow species *(Salix* L.) used in this study. We indicated species name (according in Argus [4] and IPNI), their status in America (native or introduced), their principal native area, their subgenus (Argus [4]), the herbarium informations : live collections of the Montreal Botanical Garden (MBG), the Canadian Museum of Nature (CAN), the Herbarium of the University of Texas (TEX), the University of Arizona Herbarium (ARIZ), the Missouri Botanical Garden Herbarium (MO), or unmounted. Finally, we have indicated the GenBank number. | | | | | | | | |
| --- | --- | --- | --- | --- | --- | --- | --- | --- |
|  |  |  |  |  |  |  |  |  |
| **ID used in this study** | **Species** | **Status in America** | **Principal native area (Argus [3]; personnal communication)** | **Subgenus (Argus [4])** | **Herbarium** | **Genbank accessions ID** | | |
|  |  |  |  |  |  | **ITS** | **matK** | **rbcL** |
| ALM_salix_0001 | *Salix aeruginosa* E.Carranza | Native | Mexico | Protitea | CAN | SX37100 | SX47100 | SX57100 |
| ALM_salix_0006 | *S. alaxensis* (Andersson) Coville var. *longistylis* (Rydb.) C.K.Schneid. | Native | Western Boreo-Arctic | Vetrix | CAN | SX37101 | SX47101 | SX57101 |
| ALM_salix_0960 | *S. alba* L. | Introduced | Eurasia | Salix | MBG | SX37102 | SX47102 | SX57102 |
| ALM_salix_1018 |  |  |  |  | MBG | SX37103 | SX47103 | SX57103 |
| ALM_salix_1035 |  |  |  |  | MBG | SX37104 | SX47104 | SX57104 |
| ALM_salix_0010 | *S. amygdaloides* Andersson | Native | Western and Eastern Temperate | Protitea | CAN | SX37105 | SX47105 | SX57105 |
| ALM_salix_0013 | *S. arbusculoides* Andersson | Native | Western Boreo-Arctic | Vetrix | CAN | SX37106 | SX47106 | SX57106 |
| ALM_salix_2100 | *S. arbutifolia* Pall. | Introduced | - | - | MBG | SX37309 | SX47309 | SX57309 |
| ALM_salix_0019 | *S. arctica* Pall. | Native | Western and Eastern Boreo-Arctic | Chamaetia | CAN | SX37107 | SX47107 | SX57107 |
| ALM_salix_1020 | *S.* *arctophila* Cockerell in A.Heller | Native | Eastern Boreo-Artic | Chamaetia | MBG | SX37108 | SX47108 | SX57108 |
| ALM_salix_0025 | *S. argyrocarpa* Andersson | Native | Eastern Boreo-Artic | Vetrix | CAN | SX37109 | SX47109 | SX57109 |
| ALM_salix_0026 |  |  |  |  | CAN | SX37110 | SX47110 | SX57110 |
| ALM_salix_0027 | *S. arizonica* Dorn | Native | Western Temperate | Vetrix | CAN | SX37111 | SX47111 | SX57111 |
| ALM_salix_0028 | *S. athabascensis* Raup | Native | Western Boreo-Arctic | Chamaetia | CAN | SX37112 | SX47112 | SX57112 |
| ALM_salix_1419 | *S. atrocinerea* Brot. | Introduced | Europe | Vetrix | MT | - | SX47312 | SX57312 |
| ALM_salix_2419 |  |  |  |  | MT | - | SX47313 | SX57313 |
| ALM_salix_1272 | *S.* *aurita* L. | Introduced | Europe | Vetrix | MT | SX37113 | SX47113 | SX57113 |
| ALM_salix_1273 |  |  |  |  | MT | SX37114 | SX47114 | SX57114 |
| ALM_salix_0894 | *S. babylonica* L. | Introduced | Asia | Salix | MBG | SX37115 | SX47115 | SX57115 |
| ALM_salix_0967 |  |  |  |  | MBG | SX37116 | SX47116 | SX57116 |
| ALM_salix_0239 | *S. ballii* Dorn | Native | Eastern Boreo-Artic | Vetrix | unmounted | SX37117 | SX47117 | SX57117 |
| ALM_salix_0240 |  |  |  |  | unmounted | SX37118 | SX47118 | SX57118 |
| ALM_salix_0030 | *S. barclayi* Andersson | Native | Western Boreo-Arctic | Vetrix | unmounted | SX37119 | SX47119 | SX57119 |
| ALM_salix_0031 |  |  |  |  | CAN | SX37120 | SX47120 | SX57120 |
| ALM_salix_0032 |  |  |  |  | CAN | SX37121 | SX47121 | SX57121 |
| ALM_salix_0036 | *S. barrattiana* Hook. | Native | Western Boreo-Arctic | Vetrix | CAN | SX37122 | SX47122 | SX57122 |
| ALM_salix_0037 |  |  |  |  | CAN | SX37123 | SX47123 | SX57123 |
| ALM_salix_0040 | *S.* *bebbiana* Sarg. | Native | Western and Easter Boreo-Arctic | Vetrix | CAN | SX37124 | SX47124 | SX57124 |
| ALM_salix_0041 |  |  |  |  | CAN | SX37125 | SX47125 | SX57125 |
| ALM_salix_0043 | *S. bonplandiana* Kunth | Native | Western Temperate and Mexico | Protitea | CAN | SX37126 | SX47126 | SX57126 |
| ALM_salix_0044 |  |  |  |  | CAN | SX37127 | SX47127 | SX57127 |
| ALM_salix_0047 | *S. boothii* Dorn | Native | Western Temperate | Vetrix | CAN | SX37128 | SX47128 | SX57128 |
| ALM_salix_0048 |  |  |  |  | CAN | SX37129 | SX47129 | SX57129 |
| ALM_salix_0053 | *S. brachycarpa* Nutt. var. *brachycarpa* | Native | Western and Easter Boreo-Arctic | Chamaetia | CAN | SX37130 | SX47130 | SX57130 |
| ALM_salix_0054 |  |  |  |  | CAN | SX37131 | SX47131 | SX57131 |
| ALM_salix_1414 | *S. breweri* Bebb | Native | Western Temperate | Vetrix | MT | SX37132 | SX47132 | SX57132 |
| ALM_salix_1415 |  |  |  |  | MT | SX37133 | SX47133 | SX57133 |
| ALM_salix_0055 | *S. calcicola* Fernald & Wiegand var. *glandulosior* B.Boivin | Native | Western Boreo-Artic | Vetrix | CAN | SX37134 | SX47134 | SX57134 |
| ALM_salix_0056 |  |  |  |  | CAN | SX37135 | SX47135 | SX57135 |
| ALM_salix_1552 | *S. cana* M.Martens & Galeotti | Native | Mexico | Vetrix | MO | SX37136 | SX47136 | SX57136 |
| ALM_salix_0057 |  |  |  |  | CAN | SX37137 | SX47137 | SX57137 |
| ALM_salix_0061 | *S. candida* Flüggé ex Willd. | Native | Western and Easter Boreo-Arctic | Vetrix | CAN | SX37138 | SX47138 | SX57138 |
| ALM_salix_0062 |  |  |  |  | CAN | SX37139 | SX47139 | SX57139 |
| ALM_salix_1278 | *S. caprea* L. | Introduced | Europe | Vetrix | MT | SX37140 | SX47140 | SX57140 |
| ALM_salix_1279 |  |  |  |  | MT | SX37141 | SX47141 | SX57141 |
| ALM_salix_0063 | *S. caroliniana* Michx. | Native | Eastern Temperate and Central America | Protitea | CAN | SX37142 | SX47142 | SX57142 |
| ALM_salix_0064 |  |  |  |  | CAN | SX37143 | SX47143 | SX57143 |
| ALM_salix_1405 | *S. cascadensis* Cockerell | Native | Western Temperate | Chamaetia | MT | SX37144 | SX47144 | SX57144 |
| ALM_salix_0068 | *S. chamissonis* Andersson | Native | Western Boreo-Arctic | Chamaetia | unmounted | SX37145 | SX47145 | SX57145 |
| ALM_salix_0069 |  |  |  |  | unmounted | SX37146 | SX47146 | SX57146 |
| ALM_salix_0983 | *S. chlorolepis* Fernald | Native | Eastern Boreo-Artic | Chamaetia | MBG | SX37147 | SX47147 | SX57147 |
| ALM_salix_0975 |  |  |  |  | MBG | SX37148 | SX47148 | SX57148 |
| ALM_salix_1282 | *S. cinerea* L. | Introduced | Eurasia | Vetrix | MT | SX37149 | SX47149 | SX57149 |
| ALM_salix_1036 |  |  |  |  | MBG | SX37150 | SX47150 | SX57150 |
| ALM_salix_1558 | *S. columbiana* (Dorn) Argus | Native | Western Temperate | Longifoliae | CAN | SX37151 | SX47151 | SX57151 |
| ALM_salix_1559 |  |  |  |  | CAN | SX37152 | SX47152 | SX57152 |
| ALM_salix_0070 | *S. commutata* Bebb | Native | Western Boreo-Arctic | Vetrix | CAN | SX37153 | SX47153 | SX57153 |
| ALM_salix_0246 |  |  |  |  | CAN | SX37154 | SX47154 | SX57154 |
| ALM_salix_0074 | *S. cordata* Michx. | Native | Eastern Boreo-Artic | Vetrix | CAN | SX37155 | SX47155 | SX57155 |
| ALM_salix_0075 |  |  |  |  | CAN | SX37156 | SX47156 | SX57156 |
| ALM_salix_0947 | *S. daphnoides* Vill. | Introduced | Europe | Vetrix | MBG | SX37157 | SX47157 | SX57157 |
| ALM_salix_1416 | *S. delnortensis* C.K.Schneid. | Native | Western Temperate | Vetrix | MT | SX37158 | SX47158 | SX57158 |
| ALM_salix_1417 |  |  |  |  | MT | SX37159 | SX47159 | SX57159 |
| ALM_salix_0076 | *S. discolor* Muhl. | Native | Western and Easter Boreo-Arctic | Vetrix | CAN | SX37160 | SX47160 | SX57160 |
| ALM_salix_0077 |  |  |  |  | CAN | SX37161 | SX47161 | SX57161 |
| ALM_salix_0080 | *S. drummondiana* Barratt | Native | Western Temperate | Vetrix | CAN | SX37162 | SX47162 | SX57162 |
| ALM_salix_0081 |  |  |  |  | CAN | SX37163 | SX47163 | SX57163 |
| ALM_salix_0085 | *S. eastwoodiae* Cockerell ex A.Heller | Native | Western Temperate | Vetrix | CAN | SX37164 | SX47164 | SX57164 |
| ALM_salix_0946 | *S. elaeagnos* Scop. | Introduced | Europe | Vetrix | MBG | SX37165 | SX47165 | SX57165 |
| ALM_salix_1289 |  |  |  |  | MT | SX37166 | SX47166 | SX57166 |
| ALM_salix_0087 | *S. eriocephala* Michx. | Native | Eastern Temperate | Vetrix | CAN | SX37167 | SX47167 | SX57167 |
| ALM_salix_0088 |  |  |  |  | CAN | SX37168 | SX47168 | SX57168 |
| ALM_salix_0089 |  |  |  |  | CAN | SX37169 | SX47169 | SX57169 |
| ALM_salix_0916 | *S. euxina* I.V.Belyaeva | Introduced | Asia | Salix | MBG | SX37170 | SX47170 | SX57170 |
| ALM_salix_0091 | *S. exigua* Nutt. var. *exigua* | Native | Western Temperate and Mexico | Longifoliae | CAN | SX37171 | SX47171 | SX57171 |
| ALM_salix_0092 |  |  |  |  | CAN | SX37172 | SX47172 | SX57172 |
| ALM_salix_0096 | *S. famelica* (C.R.Ball) Argus | Native | Western Boreo-Arctic | Vetrix | CAN | SX37173 | SX47173 | SX57173 |
| ALM_salix_0097 |  |  |  |  | CAN | SX37174 | SX47174 | SX57174 |
| ALM_salix_0098 | *S. farriae* C.R.Ball | Native | Western Boreo-Arctic | Vetrix | CAN | SX37175 | SX47175 | SX57175 |
| ALM_salix_0100 | *S. floridana* Chapm. | Native | Eastern Temperate | Protitea | CAN | SX37176 | SX47176 | SX57176 |
| ALM_salix_0101 |  |  |  |  | CAN | SX37177 | SX47177 | SX57177 |
| ALM_salix_0102 | *S. fuscescens* Andersson | Native | Western Boreo-Arctic | Chamaetia | unmounted | SX37178 | SX47178 | SX57178 |
| ALM_salix_0103 |  |  |  |  | unmounted | SX37179 | SX47179 | SX57179 |
| ALM_salix_1292 |  |  |  |  | MT | SX37180 | SX47180 | SX57180 |
| ALM_salix_0104 | *S. geyeriana* Andersson | Native | Western Temperate | Vetrix | CAN | SX37181 | SX47181 | SX57181 |
| ALM_salix_0105 |  |  |  |  | CAN | SX37182 | SX47182 | SX57182 |
| ALM_salix_0106 | *S. glauca* L. var. *villosa* Andersson | Native | Western and Easter Boreo-Arctic | Chamaetia | CAN | SX37183 | SX47183 | SX57183 |
| ALM_salix_0107 |  |  |  |  | CAN | SX37184 | SX47184 | SX57184 |
| ALM_salix_1294 |  |  |  |  | MT | SX37185 | SX47185 | SX57185 |
| ALM_salix_0116 | *S. gooddingii* C.R.Ball | Native | Western Temperate and Mexico | Protitea | CAN | SX37186 | SX47186 | SX57186 |
| ALM_salix_0117 |  |  |  |  | CAN | SX37187 | SX47187 | SX57187 |
| ALM_salix_1300 | *S. hastata* L. | Native | Western Boreo-Arctic | Vetrix | MT | SX37188 | SX47188 | SX57188 |
| ALM_salix_1302 |  |  |  |  | MT | SX37189 | SX47189 | SX57189 |
| ALM_salix_1180 | *S. herbacea* L. | Native | Eastern Boreo-Artic | Chamaetia | MT | SX37190 | SX47190 | SX57190 |
| ALM_salix_1181 |  |  |  |  | MT | SX37191 | SX47191 | SX57191 |
| ALM_salix_0248 | *S. hookeriana* Barratt ex Hook. | Native | Western Temperate | Vetrix | unmounted | SX37192 | SX47192 | SX57192 |
| ALM_salix_0249 |  |  |  |  | CAN | SX37193 | SX47193 | SX57193 |
| ALM_salix_0120 | *S. humboldtiana* Willd. | Native | Mexico and Central-South America | Protitea | CAN | SX37194 | SX47194 | SX57194 |
| ALM_salix_0121 |  |  |  |  | CAN | SX37195 | SX47195 | SX57195 |
| ALM_salix_0123 | *S. humilis* Marshall var. *humilis* | Native | Eastern Temperate | Vetrix | CAN | SX37196 | SX47196 | SX57196 |
| ALM_salix_0124 |  |  |  |  | CAN | SX37197 | SX47197 | SX57197 |
| ALM_salix_0126 | *S. interior* Rowlee | Native | Western and Easter Temperate and Mexico | Longifoliae | CAN | SX37198 | SX47198 | SX57198 |
| ALM_salix_1003 | *S. irrorata* Andersson | Native | Western Temperate and Mexico | Vetrix | MBG | SX37199 | SX47199 | SX57199 |
| ALM_salix_1111 |  |  |  |  | MBG | SX37200 | SX47200 | SX57200 |
| ALM_salix_1556 | *S. jaliscana* M.E.Jones | Native | Mexico | Protitea | TEX | SX37201 | SX47201 | SX57201 |
| ALM_salix_0868 | *S. jejuna* Fernald | Native | Eastern Boreo-Artic | Chamaetia | MBG | SX37202 | SX47202 | SX57202 |
| ALM_salix_0133 | *S. jepsonii* C.K.Schneid. | Native | Western Temperate | Vetrix | CAN | SX37203 | SX47203 | SX57203 |
| ALM_salix_0134 |  |  |  |  | CAN | SX37204 | SX47204 | SX57204 |
| ALM_salix_0136 | *S. laevigata* Bebb | Native | Western Temperate and Mexico | Protitea | CAN | SX37205 | SX47205 | SX57205 |
| ALM_salix_0137 | *S. lasiandra* Benth. var. *caudata* | Native | Western Temperate | Salix | CAN | SX37206 | SX47206 | SX57206 |
| ALM_salix_0138 | *S. lasiandra* Benth. var. *lasiandra* (Nutt.) Sudw. |  | Western Temperate | Salix | CAN | SX37207 | SX47207 | SX57207 |
| ALM_salix_0140 | *S. lasiolepis* Benth. | Native | Western Temperate and Mexico | Vetrix | CAN | SX37208 | SX47208 | SX57208 |
| ALM_salix_0141 |  |  |  |  | CAN | SX37209 | SX47209 | SX57209 |
| ALM_salix_0143 | *S. lemmonii* Bebb | Native | Western Temperate | Vetrix | CAN | SX37210 | SX47210 | SX57210 |
| ALM_salix_0144 |  |  |  |  | CAN | SX37211 | SX47211 | SX57211 |
| ALM_salix_0145 | *S. ligulifolia* C.R.Ball ex C.K.Schneid. | Native | Western Temperate | Vetrix | CAN | SX37212 | SX47212 | SX57212 |
| ALM_salix_0146 | *S. lucida* Muhl. | Native | Eastern Boreo-Arctic | Salix | CAN | SX37213 | SX47213 | SX57213 |
| ALM_salix_0148 | *S. lutea* Nutt. | Native | Western Temperate | Vetrix | CAN | SX37214 | SX47214 | SX57214 |
| ALM_salix_0149 |  |  |  |  | CAN | SX37215 | SX47215 | SX57215 |
| ALM_salix_0150 | *S. maccalliana* Rowlee | Native | Western Boreo-Arctic | Salix | CAN | SX37216 | SX47216 | SX57216 |
| ALM_salix_1403 | *S. melanopsis* Nutt. | Native | Western Temperate | Longifoliae | MT | SX37217 | SX47217 | SX57217 |
| ALM_salix_1404 |  |  |  |  | MT | SX37218 | SX47218 | SX57218 |
| ALM_salix_2000 | *S. mexicana* Seemen | Native | Mexico | Vetrix | ARIZ | SX37219 | SX47219 | SX57219 |
| ALM_salix_0151 | *S. microphylla* Schldl. & Cham. | Native | Mexico and Central America | Longifoliae | CAN | SX37220 | SX47220 | SX57220 |
| ALM_salix_0152 |  |  |  |  | CAN | SX37221 | SX47221 | SX57221 |
| ALM_salix_1408 | *S. monochroma* C.R.Ball | Native | Western Temperate | Vetrix | MT | SX37223 | SX47223 | SX57223 |
| ALM_salix_1409 |  |  |  |  | MT | SX37224 | SX47224 | SX57224 |
| ALM_salix_0153 | *S. monticola* Bebb | Native | Western Temperate | Vetrix | CAN | SX37225 | SX47225 | SX57225 |
| ALM_salix_0154 | *S. myricoides* Muhl. | Native | Eastern Boreo-Arctic | Vetrix | CAN | SX37226 | SX47226 | SX57226 |
| ALM_salix_0155 |  |  |  |  | CAN | SX37227 | SX47227 | SX57227 |
| ALM_salix_1096 | *S. myrsinifolia* Salisb. | Introduced | Eurasia | Vetrix | MBG | SX37228 | SX47228 | SX57228 |
| ALM_salix_1199 |  |  |  |  | MT | SX37229 | SX47229 | SX57229 |
| ALM_salix_0158 | *S. myrtillifolia* Andersson | Native | Western Boreo-Arctic | Vetrix | CAN | SX37230 | SX47230 | SX57230 |
| ALM_salix_0159 |  |  |  |  | CAN | SX37231 | SX47231 | SX57231 |
| ALM_salix_0160 | *S. nigra* Marshall | Native | Eastern Temperate and Mexico | Protitea | CAN | SX37232 | SX47232 | SX57232 |
| ALM_salix_0164 | *S. niphoclada* Rydb. | Native | Western Boreo-Arctic | Chamaetia | CAN | SX37233 | SX47233 | SX57233 |
| ALM_salix_0165 |  |  |  |  | CAN | SX37234 | SX47234 | SX57234 |
| ALM_salix_0166 | *S. nivalis* Hook. | Native | Western Temperate | Chamaetia | CAN | SX37235 | SX47235 | SX57235 |
| ALM_salix_1211 | *S. nummularia* Andersson | Native | Western Boreo-Arctic | Chamaetia | MT | SX37236 | SX47236 | SX57236 |
| ALM_salix_1410 | *S. orestera* C.K.Schneid. | Native | Western Temperate | Vetrix | MT | SX37237 | SX47237 | SX57237 |
| ALM_salix_0167 | *S. ovalifolia* Trautv. var. *ovalifolia* | Native | Western Boreo-Arctic | Chamaetia | CAN | SX37238 | SX47238 | SX57238 |
| ALM_salix_0168 | *S. paradoxa* Kunth | Native | Mexico | Vetrix | CAN | SX37239 | SX47239 | SX57239 |
| ALM_salix_0169 |  |  |  |  | CAN | SX37240 | SX47240 | SX57240 |
| ALM_salix_0170 | *S. pedicellaris* Pursh | Native | Western and Easter Boreo-Arctic | Chamaetia | CAN | SX37241 | SX47241 | SX57241 |
| ALM_salix_1081 | *S. pellita* (Andersson) Bebb | Native | Eastern Boreo-Artic | Vetrix | MBG | SX37242 | SX47242 | SX57242 |
| ALM_salix_1125 |  |  |  |  | MBG | SX37243 | SX47243 | SX57243 |
| ALM_salix_1338 | *S. pentandra* L. | Introduced | Eurasia | Salix | MT | SX37244 | SX47244 | SX57244 |
| ALM_salix_1339 |  |  |  |  | MT | SX37245 | SX47245 | SX57245 |
| ALM_salix_0174 | *S. petiolaris* Sm. | Native | Western and Easter Boreo-Arctic | Vetrix | CAN | SX37246 | SX47246 | SX57246 |
| ALM_salix_0951 |  |  |  |  | MBG | SX37247 | SX47247 | SX57247 |
| ALM_salix_0175 | *S. petrophila* Rydb. | Native | Western Temperate | Chamaetia | CAN | SX37248 | SX47248 | SX57248 |
| ALM_salix_0176 | *S. phlebophylla* Andersson | Native | Western Boreo-Arctic | Chamaetia | unmounted | SX37249 | SX47249 | SX57249 |
| ALM_salix_0177 |  |  |  |  | unmounted | SX37250 | SX47250 | SX57250 |
| ALM_salix_0178 | *S. planifolia* Pursh | Native | Western and Easter Boreo-Arctic | Vetrix | CAN | SX37251 | SX47251 | SX57251 |
| ALM_salix_0179 |  |  |  |  | CAN | SX37252 | SX47252 | SX57252 |
| ALM_salix_0181 | *S. polaris* Wahlenb. | Native | Western Boreo-Arctic | Chamaetia | CAN | SX37253 | SX47253 | SX57253 |
| ALM_salix_1413 | *S. prolixa* Andersson | Native | Western Temperate-Boreo-Arctic | Vetrix | MT | SX37254 | SX47254 | SX57254 |
| ALM_salix_0182 | *S. pseudomonticola* C.R.Ball | Native | Western Boreo-Arctic | Vetrix | CAN | SX37310 | SX47310 | SX57310 |
| ALM_salix_0183 |  |  |  |  | CAN | SX37311 | SX47311 | SX57311 |
| ALM_salix_0185 | *S. pseudomyrsinites* Andersson | Native | Western Boreo-Arctic | Vetrix | CAN | SX37255 | SX47255 | SX57255 |
| ALM_salix_0186 |  |  |  |  | CAN | SX37256 | SX47256 | SX57256 |
| ALM_salix_0188 | *S. pulchra* Cham. | Native | Western Boreo-Arctic | Vetrix | CAN | SX37257 | SX47257 | SX57257 |
| ALM_salix_0190 |  |  |  |  | CAN | SX37258 | SX47258 | SX57258 |
| ALM_salix_1421 | *S. purpurea* L. | Introduced | Europe | Vetrix | MT | SX37259 | SX47259 | SX57259 |
| ALM_salix_1422 |  |  |  |  | MT | SX37260 | SX47260 | SX57260 |
| ALM_salix_1423 |  |  |  |  | MT | SX37261 | SX47261 | SX57261 |
| ALM_salix_0191 | *S. pyrifolia* Andersson | Native | Western and Easter Boreo-Arctic | Vetrix | CAN | SX37262 | SX47262 | SX57262 |
| ALM_salix_0192 |  |  |  |  | CAN | SX37263 | SX47263 | SX57263 |
| ALM_salix_1560 | *S. raupii* Argus | Native | Western Boreo-Arctic | Chamaetia | CAN | SX37264 | SX47264 | SX57264 |
| ALM_salix_1561 |  |  |  |  | CAN | SX37265 | SX47265 | SX57265 |
| ALM_salix_1562 |  |  |  |  | CAN | SX37266 | SX47266 | SX57266 |
| ALM_salix_0193 | *S. reticulata* L. | Native | Western and Easter Boreo-Arctic | Chamaetia | CAN | SX37267 | SX47267 | SX57267 |
| ALM_salix_0194 |  |  |  |  | CAN | SX37268 | SX47268 | SX57268 |
| ALM_salix_0198 | *S. richardsonii* Hook. | Native | Western Boreo-Arctic | Vetrix | CAN | SX37269 | SX47269 | SX57269 |
| ALM_salix_0199 |  |  |  |  | CAN | SX37270 | SX47270 | SX57270 |
| ALM_salix_1557 | *S. riskindii* M.C.Johnst. | Native | Mexico | Vetrix | TEX | SX37271 | SX47271 | SX57271 |
| ALM_salix_0205 | *S. rotundifolia* Trautv. var. *dodgeana* (Rydb.) A.E.Murray | Native | Western Boreo-Arctic | Chamaetia | CAN | SX37272 | SX47272 | SX57272 |
| ALM_salix_0206 |  |  |  |  | CAN | SX37273 | SX47273 | SX57273 |
| ALM_salix_0210 | *S. scouleriana* Barratt ex Hook. | Native | Western Temperate-Boreo-Arctic and Mexico | Vetrix | CAN | SX37274 | SX47274 | SX57274 |
| ALM_salix_0211 |  |  |  |  | CAN | SX37275 | SX47275 | SX57275 |
| ALM_salix_0213 | *S. sericea* Marshall | Native | Eastern Temperate | Vetrix | CAN | SX37276 | SX47276 | SX57276 |
| ALM_salix_0214 |  |  |  |  | CAN | SX37277 | SX47277 | SX57277 |
| ALM_salix_0215 | *S. serissima* (L.H.Bailey) Fernald | Native | Western and Easter Boreo-Arctic | Salix | CAN | SX37278 | SX47278 | SX57278 |
| ALM_salix_0216 |  |  |  |  | CAN | SX37279 | SX47279 | SX57279 |
| ALM_salix_1566 | *S. sessilifolia* Nutt. | Native | Western Temperate | Longifoliae | CAN | SX37280 | SX47280 | SX57280 |
| ALM_salix_0217 | *S. setchelliana* C.R.Ball | Native | Western Boreo-Arctic | Chamaetia | CAN | SX37281 | SX47281 | SX57281 |
| ALM_salix_0218 |  |  |  |  | CAN | SX37282 | SX47282 | SX57282 |
| ALM_salix_0219 |  |  |  |  | CAN | SX37283 | SX47283 | SX57283 |
| ALM_salix_0220 | *S. silicicola* Raup | Native | Western Boreo-Arctic | Vetrix | CAN | SX37284 | SX47284 | SX57284 |
| ALM_salix_0221 |  |  |  |  | CAN | SX37285 | SX47285 | SX57285 |
| ALM_salix_0222 |  |  |  |  | CAN | SX37286 | SX47286 | SX57286 |
| ALM_salix_0259 | *S. sitchensis* Sanson ex Bong. | Native | Western Temperate | Vetrix | CAN | SX37287 | SX47287 | SX57287 |
| ALM_salix_1563 | *S. sphenophylla* A.K.Skvortsov | Native | Western Boreo-Arctic | Chamaetia | CAN | SX37288 | SX47288 | SX57288 |
| ALM_salix_1564 |  |  |  |  | CAN | SX37289 | SX47289 | SX57289 |
| ALM_salix_0223 | *S. stolonifera* Coville | Native | Western Boreo-Arctic | Chamaetia | CAN | SX37290 | SX47290 | SX57290 |
| ALM_salix_0260 |  |  |  |  | unmounted | SX37291 | SX47291 | SX57291 |
| ALM_salix_0224 | *S. taxifolia* Kunth | Native | Western Temperate and Mexico | Longifoliae | CAN | SX37292 | SX47292 | SX57292 |
| ALM_salix_0225 |  |  |  |  | CAN | SX37293 | SX47293 | SX57293 |
| ALM_salix_0227 | *S. thurberi* Rowlee | Native | Western Temperate and Mexico | Longifoliae | CAN | SX37294 | SX47294 | SX57294 |
| ALM_salix_1412 | *S. tracyi* C.R.Ball | Native | Western Temperate | Vetrix | MT | SX37295 | SX47295 | SX57295 |
| ALM_salix_1082 | *S. triandra* L. | Introduced | Eurasia | Salix | MBG | SX37296 | SX47296 | SX57296 |
| ALM_salix_0228 | *S. turnorii* Raup | Native | Western Boreo-Arctic | Vetrix | unmounted | SX37297 | SX47297 | SX57297 |
| ALM_salix_0229 |  |  |  |  | unmounted | SX37298 | SX47298 | SX57298 |
| ALM_salix_1411 | *S. tweedyi* (Bebb ex Rose) C.R.Ball | Native | Western Temperate | Vetrix | MT | SX37299 | SX47299 | SX57299 |
| ALM_salix_0230 | *S. tyrrellii* Raup | Native | Western Boreo-Arctic | Vetrix | CAN | SX37300 | SX47300 | SX57300 |
| ALM_salix_0231 |  |  |  |  | unmounted | SX37301 | SX47301 | SX57301 |
| ALM_salix_0262 | *S. uva-ursi* Pursh | Native | Eastern Boreo-Artic | Chamaetia | unmounted | SX37302 | SX47302 | SX57302 |
| ALM_salix_0263 |  |  |  |  | unmounted | SX37303 | SX47303 | SX57303 |
| ALM_salix_1390 | *S. vestita* Pursh | Native | Western and Easter Boreo-Arctic | Chamaetia | MT | SX37304 | SX47304 | SX57304 |
| ALM_salix_1100 | *S. viminalis* L. | Introduced | Europe | Vetrix | MBG | SX37305 | SX47305 | SX57305 |
| ALM_salix_1253 |  |  |  |  | MT | SX37306 | SX47306 | SX57306 |
| ALM_salix_1406 | *S. wolfii* Bebb | Native | Western Temperate | Vetrix | MT | SX37307 | SX47307 | SX57307 |
| ALM_salix_1407 |  |  |  |  | MT | SX37308 | SX47308 | SX57308 |
